# Supplementary material for: Synaptic vesicle proteins are selectively delivered to axons in mammalian neurons
Source: eLife. 2023 Feb 2;12:e82568. doi: 10.7554/eLife.82568 (PMC9894587; doi:10.7554/eLife.82568)
Supplement: Figure 2—source data 1. — (A) Corresponds to Figure 2H and Figure 2I. (B) Corresponds to Figure 2J and Figure 2K. [file elife-82568-fig2-data1.docx]

**Supplementary File 1**

(**A**)

|  | SYT1 reporter | | | |  | SYB2 reporter | | | |
| --- | --- | --- | --- | --- | --- | --- | --- | --- | --- |
|  | Proximal axon | Distal axon | Proximal dendrite | Distal dendrite |  | Proximal axon | Distal axon | Proximal dendrite | Distal dendrite |
| Number of values (transport vesicles) | 29 | 21 | 4 | 6 |  | 26 | 27 | 10 | 6 |
|  |  |  |  |  |  |  |  |  |  |
| Mean | 6.27 | 10.1 | -2.33 | 1.01 |  | 3.90 | 4.27 | -0.0789 | 0.824 |
| Median | 8.69 | 11.7 | -1.49 | 0.255 |  | 4.60 | 4.60 | 0.438 | 0.725 |
| Std. Deviation | 9.4 | 8.4 | 3.6 | 2.8 |  | 9.0 | 8.7 | 8.0 | 5.9 |
| Std. Error of Mean | 1.7 | 1.8 | 1.8 | 1.1 |  | 1.8 | 1.7 | 2.5 | 2.4 |
|  |  |  |  |  |  |  |  |  |  |
| Lower 95% CI of mean | 2.70 | 6.21 | -8.06 | -1.90 |  | 0.248 | 0.839 | -5.81 | -5.36 |
| Upper 95% CI of mean | 9.84 | 13.9 | 3.39 | 3.92 |  | 7.55 | 7.70 | 5.66 | 7.00 |

(**B**)

|  | SYT1 reporter | | | |  | SYB2 reporter | | | |
| --- | --- | --- | --- | --- | --- | --- | --- | --- | --- |
|  | Proximal axon | Distal  axon | Proximal dendrite | Distal dendrite |  | Proximal axon | Distal axon | Proximal dendrite | Distal dendrite |
| Number of values (cells) | 7 | 7 | 7 | 7 |  | 7 | 7 | 7 | 7 |
|  |  |  |  |  |  |  |  |  |  |
| Mean | 4.14 | 3.00 | 0.571 | 0.857 |  | 3.71 | 3.86 | 1.43 | 0.857 |
| Median | 4.0 | 4.0 | 1.0 | 1.0 |  | 5.0 | 4.0 | 2.0 | 1.0 |
| Std. Deviation | 2.0 | 2.4 | 0.53 | 0.69 |  | 2.0 | 2.2 | 0.98 | 0.69 |
| Std. Error of Mean | 0.77 | 0.90 | 0.20 | 0.26 |  | 0.75 | 0.83 | 0.37 | 0.26 |
|  |  |  |  |  |  |  |  |  |  |
| Lower 95% CI of mean | 2.26 | 0.80 | 0.077 | 0.22 |  | 1.89 | 1.83 | 0.526 | 0.219 |
| Upper 95% CI of mean | 6.03 | 5.20 | 1.07 | 1.50 |  | 5.54 | 5.89 | 2.33 | 1.50 |
