# Supplementary material for: Synaptic vesicle proteins are selectively delivered to axons in mammalian neurons
Source: eLife. 2023 Feb 2;12:e82568. doi: 10.7554/eLife.82568 (PMC9894587; doi:10.7554/eLife.82568)
Supplement: Figure 3—source data 1. — (A) Corresponds to Figure 3B. (B) Corresponds to Figure 3C. [file elife-82568-fig3-data1.docx]

**Supplementary File 2**

(**A**)

|  | Native SYT1 | | | Native SYT1 + SYT1 reporter | | |
| --- | --- | --- | --- | --- | --- | --- |
|  | 0.1x SYT1 reporter | 1x SYT1 reporter | 10x SYT1 reporter | 0.1x SYT1 reporter | 1x SYT1 reporter | 10x SYT1 reporter |
| Number of values (synapses) | 40 | 40 | 40 | 40 | 40 | 40 |
|  |  |  |  |  |  |  |
| Mean | 0.561 | 0.400 | 0.433 | 0.641 | 0.488 | 1.074 |
| Median | 0.531 | 0.348 | 0.344 | 0.567 | 0.361 | 0.805 |
| Std. Deviation | 0.187 | 0.187 | 0.246 | 0.301 | 0.326 | 1.015 |
| Std. Error of Mean | 0.030 | 0.030 | 0.039 | 0.048 | 0.052 | 0.161 |
|  |  |  |  |  |  |  |
| Lower 95% CI of mean | 0.501 | 0.340 | 0.354 | 0.545 | 0.384 | 0.749 |
| Upper 95% CI of mean | 0.621 | 0.459 | 0.512 | 0.738 | 0.593 | 1.398 |

(**B**)

|  | Native SYB2 | | | Native SYB2 + SYB2 reporter | | |
| --- | --- | --- | --- | --- | --- | --- |
|  | 0.1x SYB2 reporter | 1x SYB2 reporter | 10x SYB2 reporter | 0.1x SYB2 reporter | 1x SYB2 reporter | 10x SYB2 reporter |
| Number of values (synapses) | 40 | 40 | 40 | 40 | 40 | 40 |
|  |  |  |  |  |  |  |
| Mean | 0.640 | 0.641 | 0.403 | 0.597 | 0.752 | 0.756 |
| Median | 0.575 | 0.494 | 0.330 | 0.554 | 0.594 | 0.653 |
| Std. Deviation | 0.239 | 0.370 | 0.197 | 0.146 | 0.542 | 0.343 |
| Std. Error of Mean | 0.038 | 0.059 | 0.031 | 0.023 | 0.086 | 0.054 |
|  |  |  |  |  |  |  |
| Lower 95% CI of mean | 0.564 | 0.523 | 0.340 | 0.551 | 0.579 | 0.646 |
| Upper 95% CI of mean | 0.717 | 0.759 | 0.467 | 0.644 | 0.925 | 0.865 |
