# Supplementary material for: Synaptic vesicle proteins are selectively delivered to axons in mammalian neurons
Source: eLife. 2023 Feb 2;12:e82568. doi: 10.7554/eLife.82568 (PMC9894587; doi:10.7554/eLife.82568)
Supplement: Figure 4—source data 1. [file elife-82568-fig4-data1.docx]

**Supplementary File 3**

| Šídák's multiple comparisons test | Mean Difference | 95% CI of difference | Below threshold? | Summary | Adjusted P Value |
| --- | --- | --- | --- | --- | --- |
| SYT1 axon vs. SYT1 dendrite | 4.50 | 0.751 to 8.25 | Yes | ** | 0.0097 |
| ΔC2AB axon vs. ΔC2AB dendrite | 2.27 | -1.30 to 5.85 | No | ns | 0.49 |
| -PGM axon vs.  -PGM dendrite | 4.38 | 0.183 to 8.57 | Yes | * | 0.036 |
| SYT1 axon vs. ΔC2AB axon | 0.691 | -2.97 to 4.35 | No | ns | >0.99 |
| -PGM axon vs. ΔC2AB axon | 0.341 | -3.55 to 4.24 | No | ns | >0.99 |
| -PGM axon vs. SYT1 axon | -0.350 | -4.33 to 3.63 | No | ns | >0.99 |
| SYT1 dendrite vs. ΔC2AB dendrite | -1.54 | -5.20 to 2.13 | No | ns | 0.907 |
| SYT1 dendrite vs.  -PGM dendrite | 0.225 | -3.75 to 4.20 | No | ns | >0.99 |
| ΔC2AB dendrite vs. -PGM dendrite | 1.76 | -2.13 to 5.66 | No | ns | 0.86 |
