# Supplementary material for: Synaptic vesicle proteins are selectively delivered to axons in mammalian neurons
Source: eLife. 2023 Feb 2;12:e82568. doi: 10.7554/eLife.82568 (PMC9894587; doi:10.7554/eLife.82568)
Supplement: Figure 4—source data 2. [file elife-82568-fig4-data2.docx]

**Supplementary File 4**

|  | SYT1 | | SYT1ΔC2AB | | SYT1-PGM | |
| --- | --- | --- | --- | --- | --- | --- |
|  | Axon | Dendrite | Axon | Dendrite | Axon | Dendrite |
| Number of values (cells) | 10 | 10 | 11 | 11 | 8 | 8 |
|  |  |  |  |  |  |  |
| Mean | 6.60 | 2.10 | 5.91 | 3.64 | 6.25 | 1.88 |
| Median | 7.5 | 1.5 | 6.0 | 3.0 | 5.5 | 1.5 |
| Std. Deviation | 4.2 | 1.8 | 3.1 | 2.3 | 3.4 | 1.8 |
| Std. Error of Mean | 1.3 | 0.57 | 0.94 | 0.70 | 1.2 | 0.64 |
|  |  |  |  |  |  |  |
| Lower 95% CI of mean | 3.62 | 0.818 | 3.82 | 2.07 | 3.43 | 0.364 |
| Upper 95% CI of mean | 9.58 | 3.38 | 8.00 | 5.21 | 9.067 | 3.39 |
