# Supplementary material for: Synaptic vesicle proteins are selectively delivered to axons in mammalian neurons
Source: eLife. 2023 Feb 2;12:e82568. doi: 10.7554/eLife.82568 (PMC9894587; doi:10.7554/eLife.82568)
Supplement: Figure 4—source data 3. [file elife-82568-fig4-data3.docx]

**Supplementary File 5**

|  | SYT1_axon_ | SYT1_dendrites_ | ΔC2AB_axon_ | ΔC2AB_dendrites_ | PGM_axon_ | PGM_dendrite_ |
| --- | --- | --- | --- | --- | --- | --- |
| anterograde | 0.52 | 0.26 | 0.43 | 0.26 | 0.40 | 0.63 |
| retrograde | 0.12 | 0.44 | 0.19 | 0.33 | 0.23 | 0.17 |
| retrograde with pause/reverse | 0.019 | 0.00 | 0.00 | 0.013 | 0.041 | 0.00 |
| anterograde with pause/reverse | 0.041 | 0.019 | 0.071 | 0.091 | 0.021 | 0.083 |
| stationary (1<5µm) | 0.067 | 0.028 | 0.077 | 0.024 | 0.00 | 0.033 |
| stationary (<1µm) | 0.24 | 0.25 | 0.23 | 0.28 | 0.31 | 0.083 |
