# Supplementary material for: Synaptic vesicle proteins are selectively delivered to axons in mammalian neurons
Source: eLife. 2023 Feb 2;12:e82568. doi: 10.7554/eLife.82568 (PMC9894587; doi:10.7554/eLife.82568)
Supplement: Figure 5—source data 1. — (A) Corresponds to Figure 5E. (B) Corresponds to Figure 5F. [file elife-82568-fig5-data1.docx]

**Supplementary File 6**

(**A**)

| SYT1 | Cultured in the absence of JF549i | Cultured in the presence of JF549i |
| --- | --- | --- |
| Number of values  (synapses) | 136 | 156 |
|  |  |  |
| Mean | 30.5 | 2.03 |
| Median | 25.4 | 1.82 |
| Std. Deviation | 21.6 | 0.925 |
| Std. Error of Mean | 1.85 | .0740 |
|  |  |  |
| Lower 95% CI of mean | 26.8 | 1.88 |
| Upper 95% CI of mean | 34.2 | 2.17 |

(**B**)

| SYB2 | Cultured in the absence of JF549i | Cultured in the presence of JF549i |
| --- | --- | --- |
| Number of values  (synapses) | 79 | 107 |
|  |  |  |
| Mean | 40.5 | 2.98 |
| Median | 36.7 | 2.89 |
| Std. Deviation | 18.0 | 1.67 |
| Std. Error of Mean | 2.03 | 0.161 |
|  |  |  |
| Lower 95% CI of mean | 36.5 | 2.66 |
| Upper 95% CI of mean | 44.6 | 3.30 |
