# Supplementary figures and images for: Synaptic vesicle proteins are selectively delivered to axons in mammalian neurons
Source: eLife. 2023 Feb 2;12:e82568. doi: 10.7554/eLife.82568 (PMC9894587; doi:10.7554/eLife.82568)

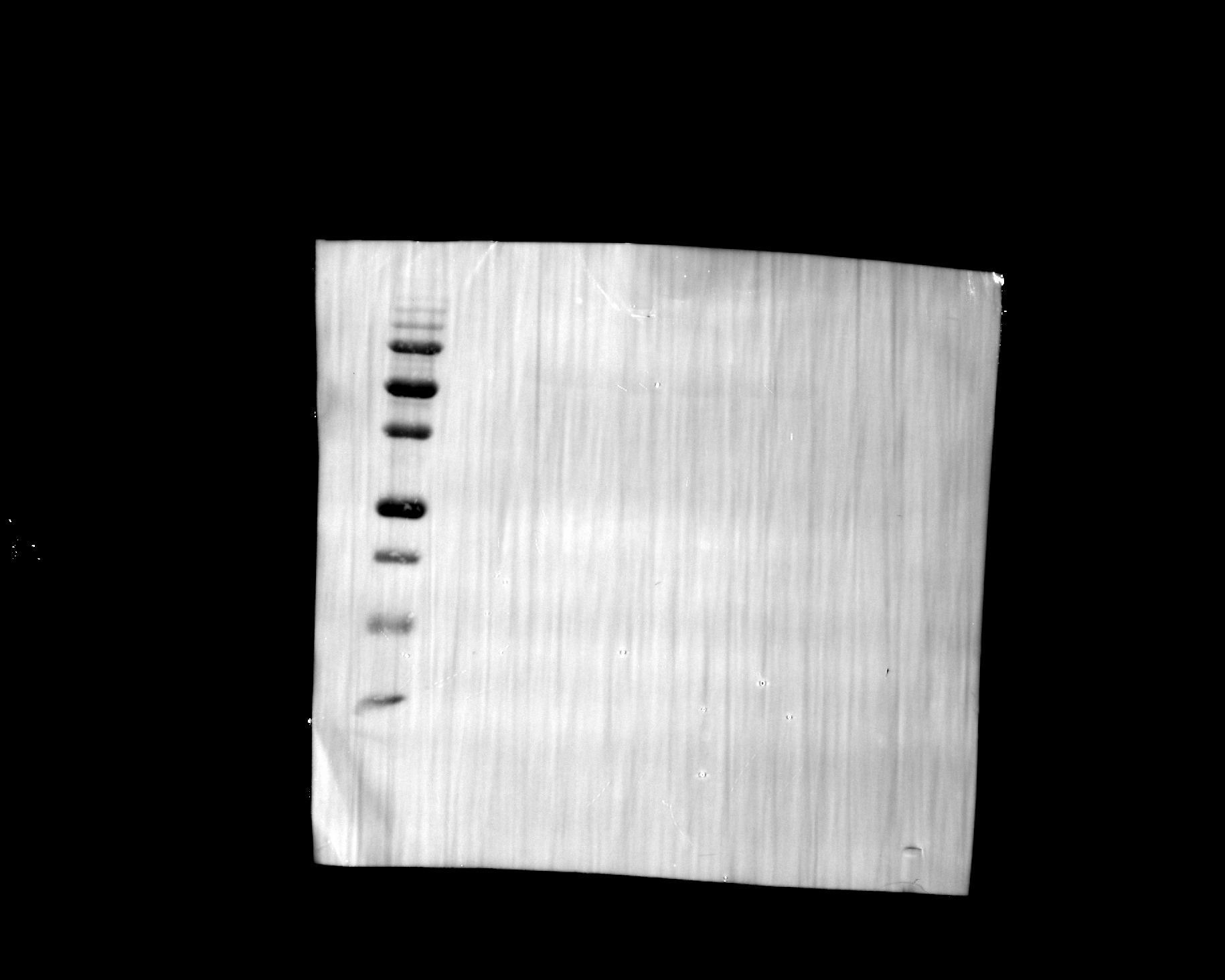

Supplement: Source data 1. [file elife-82568-data1.zip › Source Data files/Raw blot images/Figure 3 - figure supplement 1/Figure 3 - figure supplement 1A anti-SYT1 Ladder.jpg]

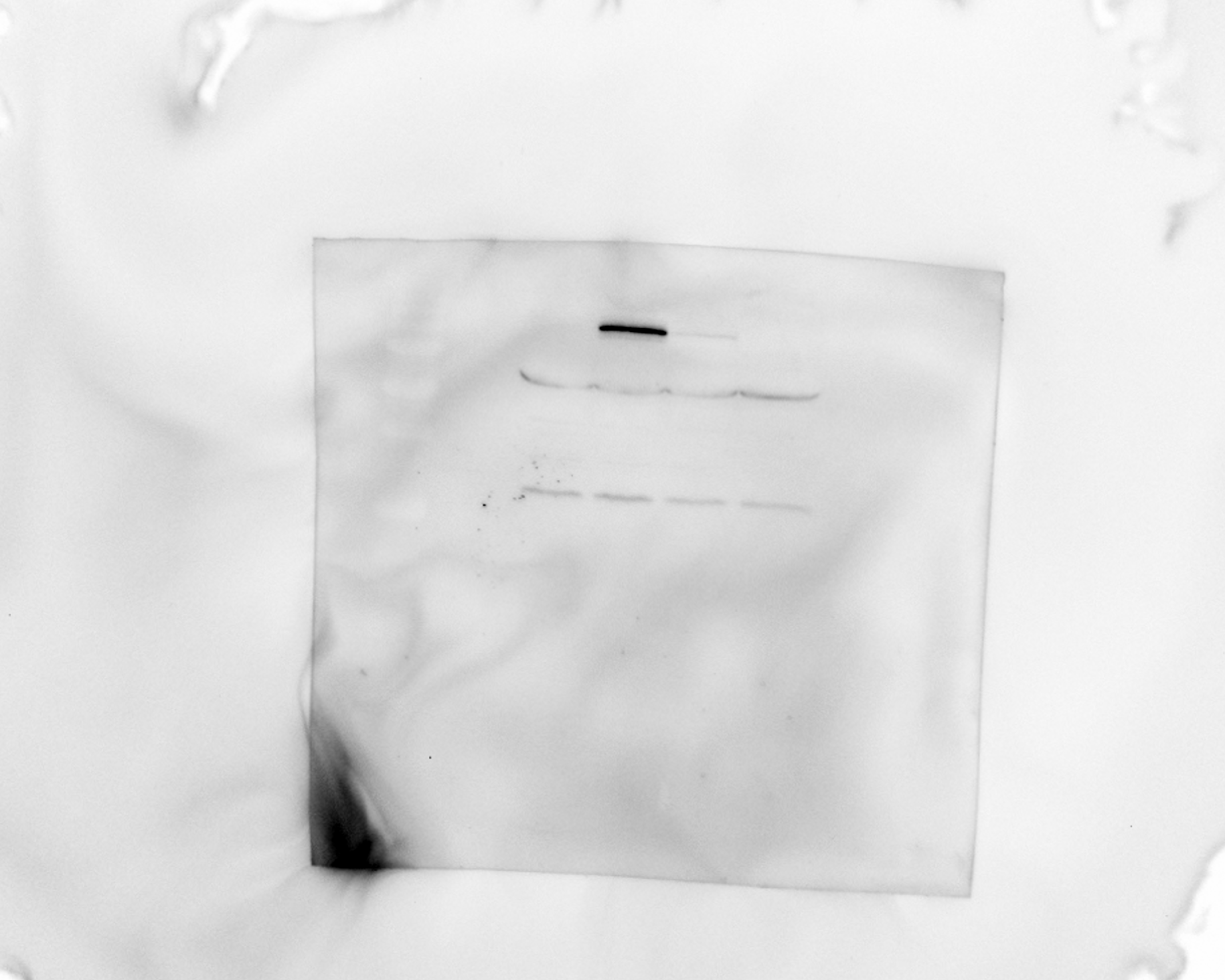

Supplement: Source data 1. [file elife-82568-data1.zip › Source Data files/Raw blot images/Figure 3 - figure supplement 1/Figure 3 - figure supplement 1A anti-SYT1.tif]

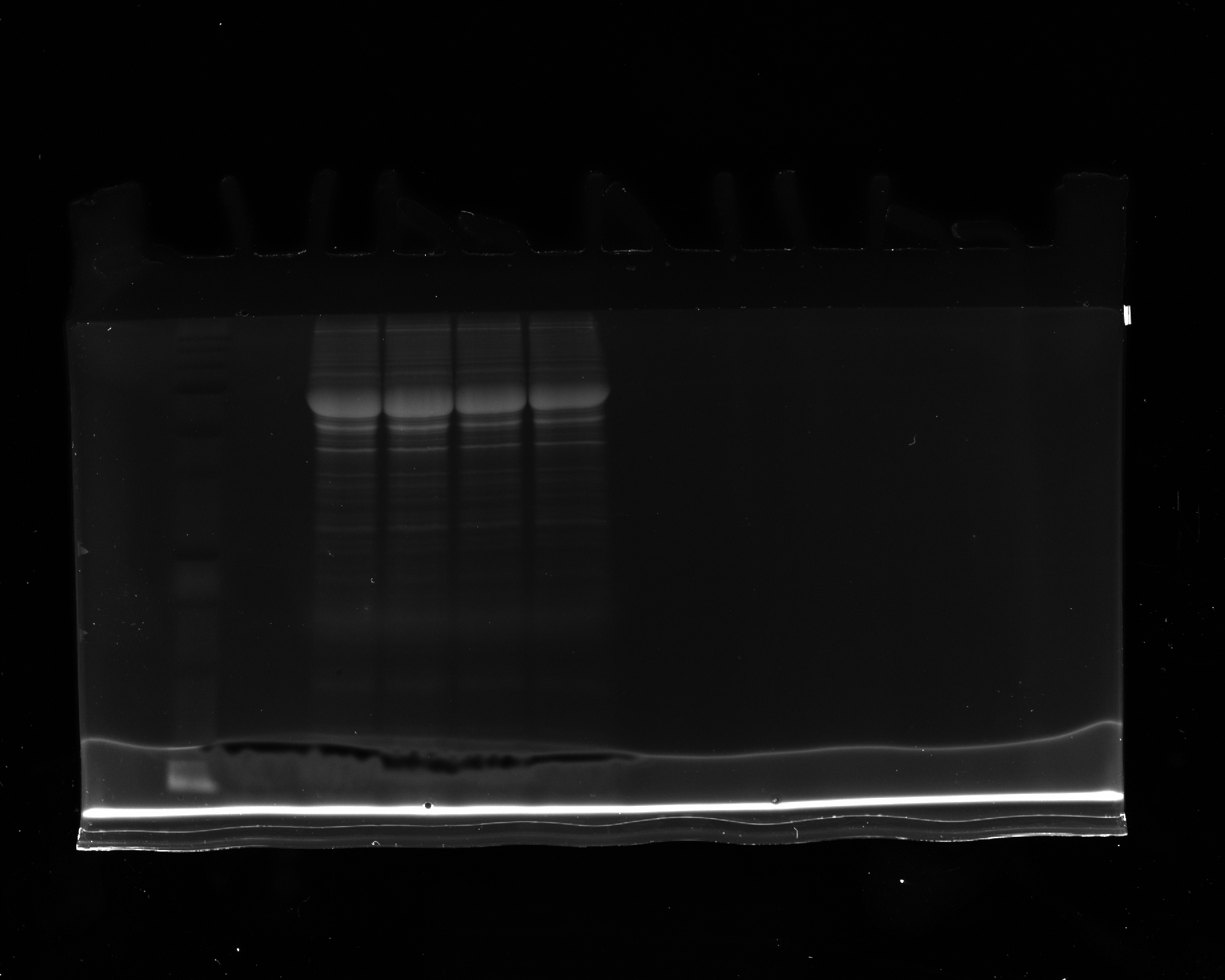

Supplement: Source data 1. [file elife-82568-data1.zip › Source Data files/Raw blot images/Figure 3 - figure supplement 1/Figure 3 - figure supplement 1A TCE loading control.jpg]

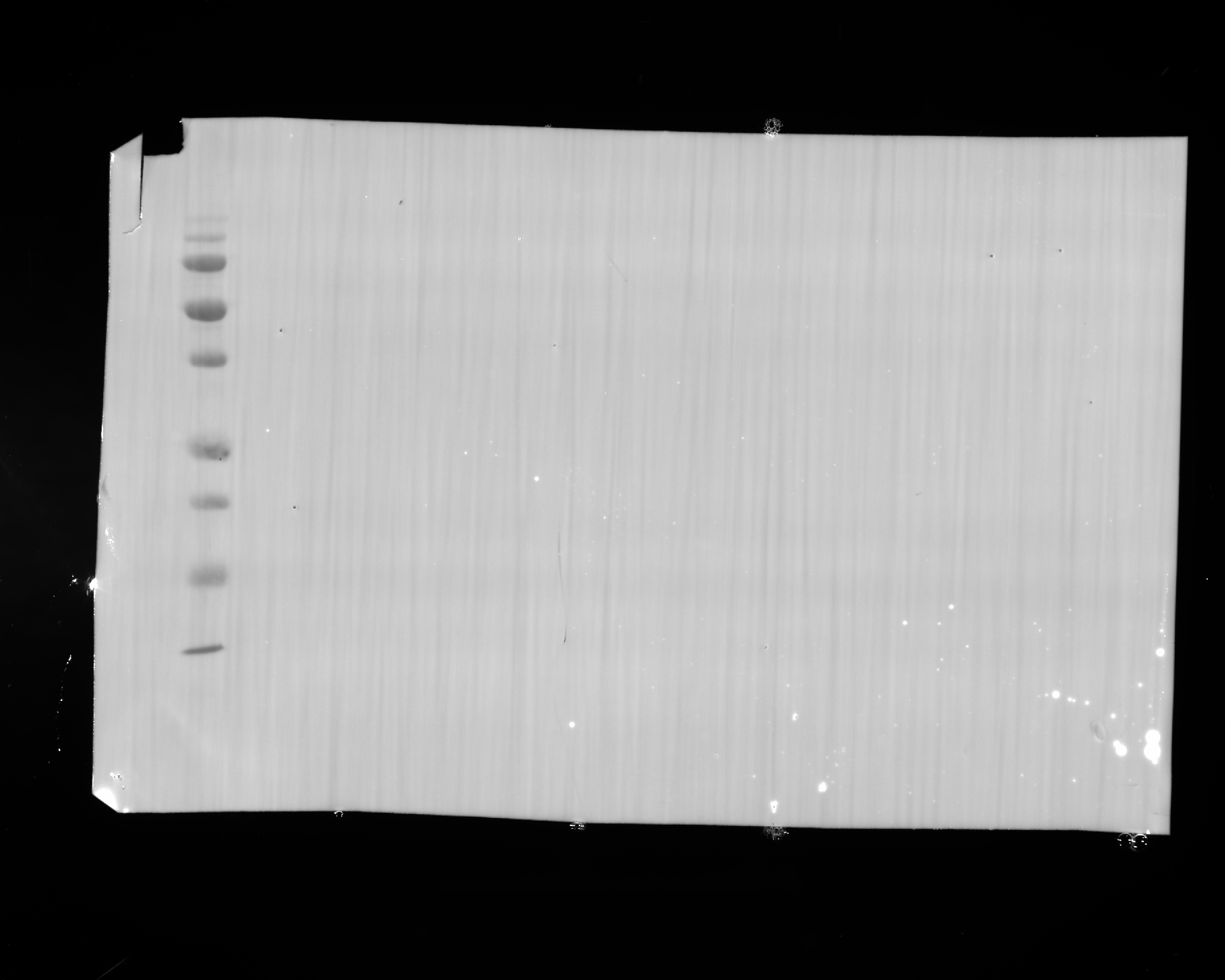

Supplement: Source data 1. [file elife-82568-data1.zip › Source Data files/Raw blot images/Figure 3 - figure supplement 1/Figure 3 - figure supplement 1B anti-SYB2 Ladder.jpg]

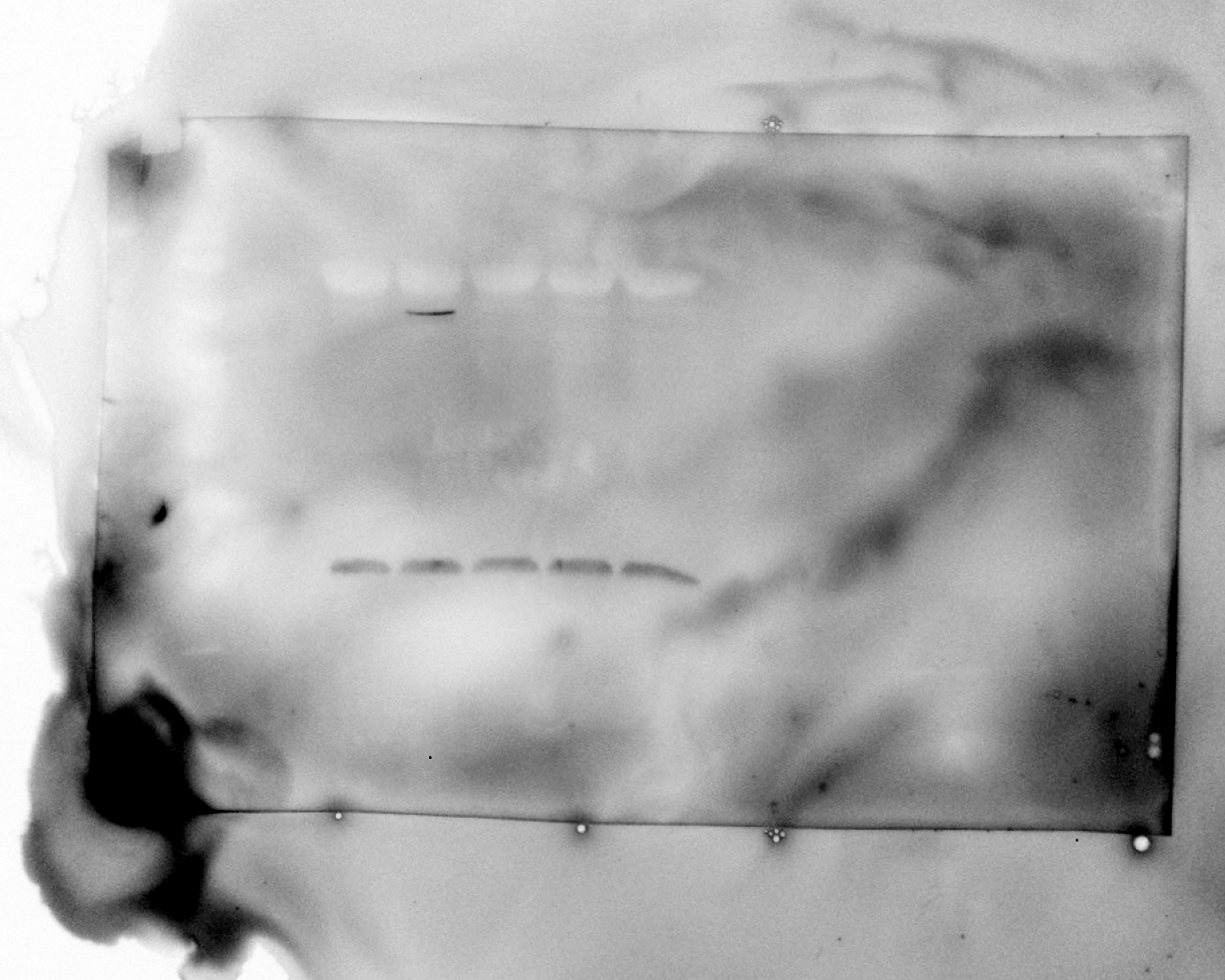

Supplement: Source data 1. [file elife-82568-data1.zip › Source Data files/Raw blot images/Figure 3 - figure supplement 1/Figure 3 - figure supplement 1B anti-SYB2.tif]

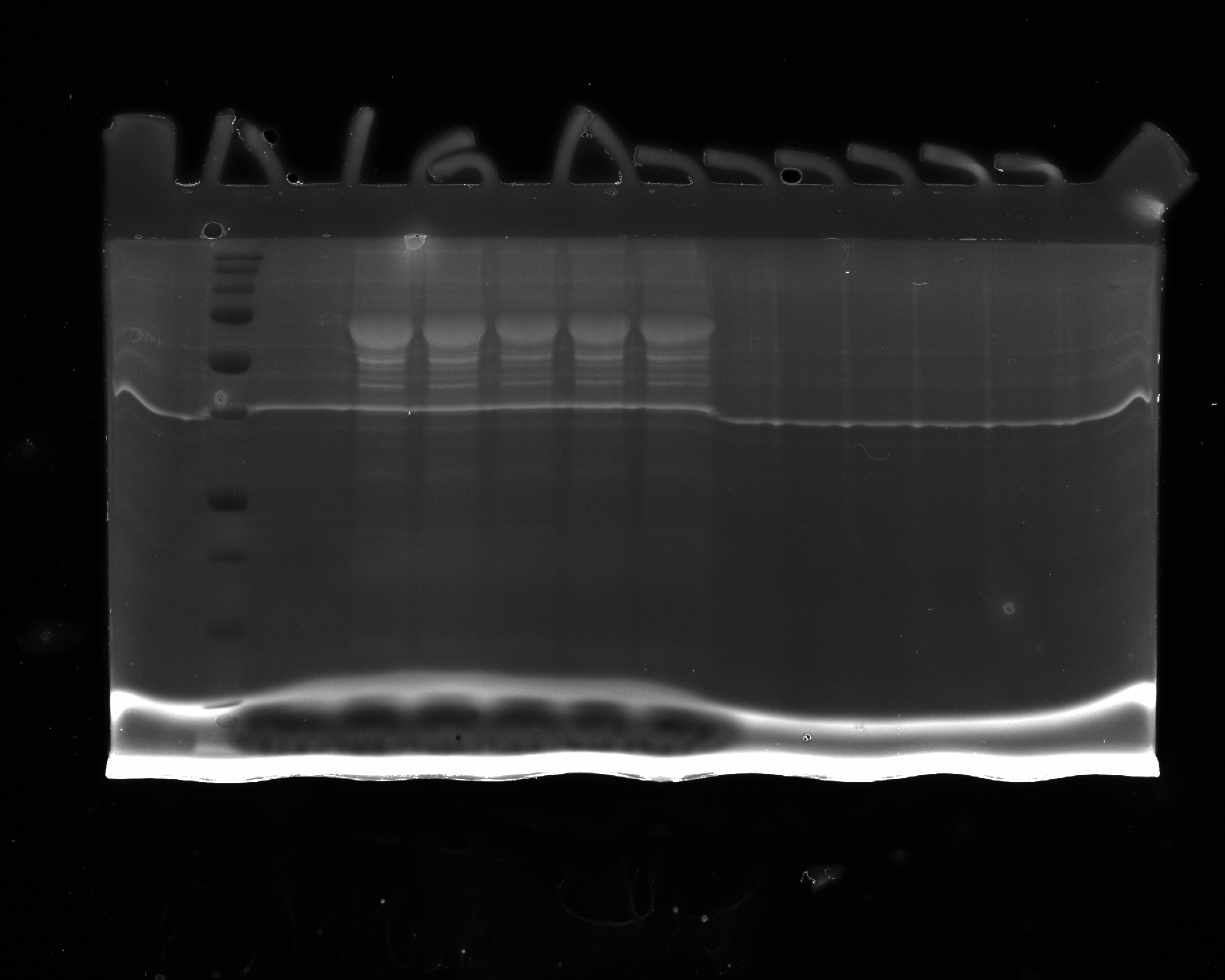

Supplement: Source data 1. [file elife-82568-data1.zip › Source Data files/Raw blot images/Figure 3 - figure supplement 1/Figure 3 - figure supplement 1B TCE loading control.jpg]

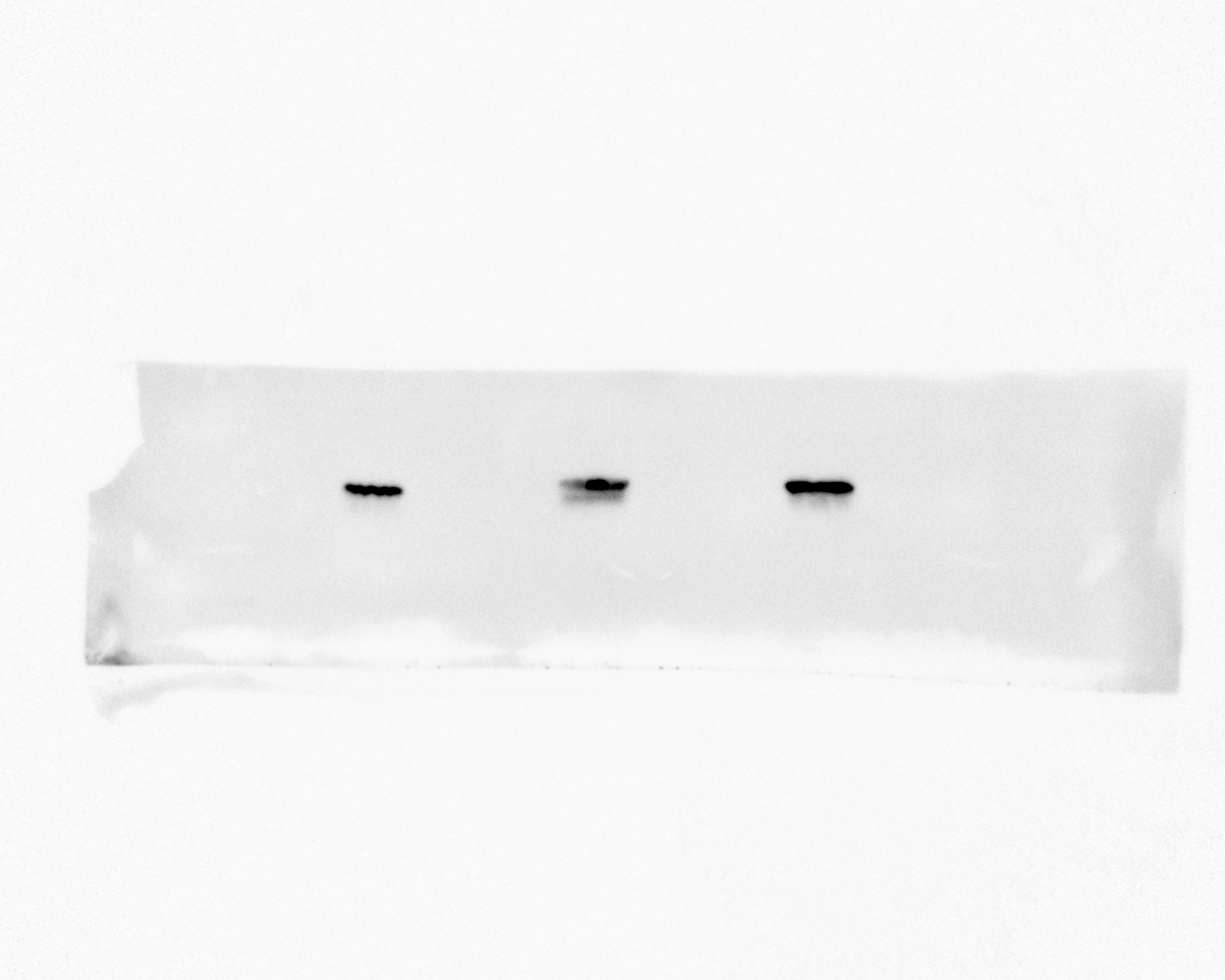

Supplement: Source data 1. [file elife-82568-data1.zip › Source Data files/Raw blot images/Figure 5/Figure 5d anti-SYB2.gif]

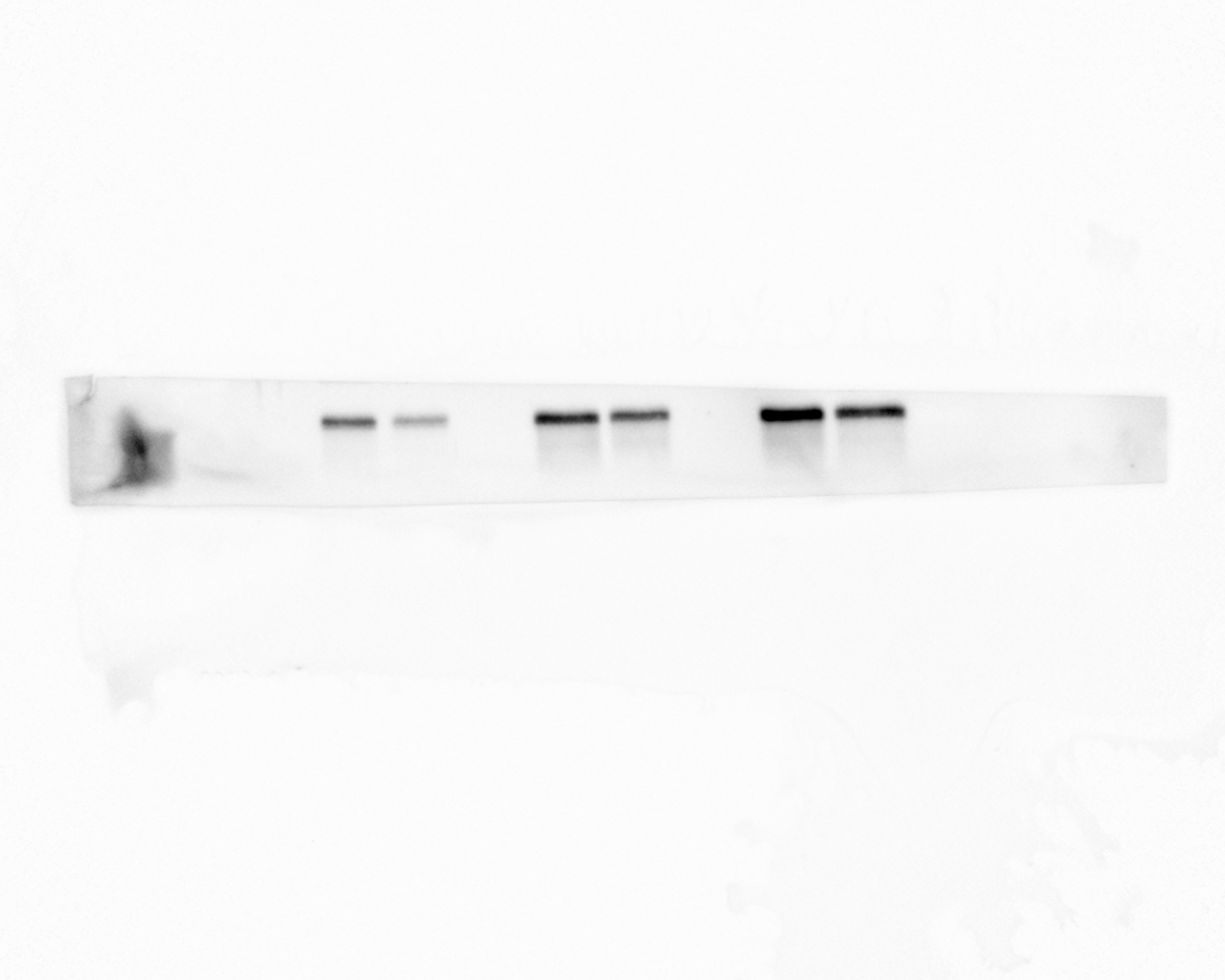

Supplement: Source data 1. [file elife-82568-data1.zip › Source Data files/Raw blot images/Figure 5/Figure 5d anti-SYP.gif]

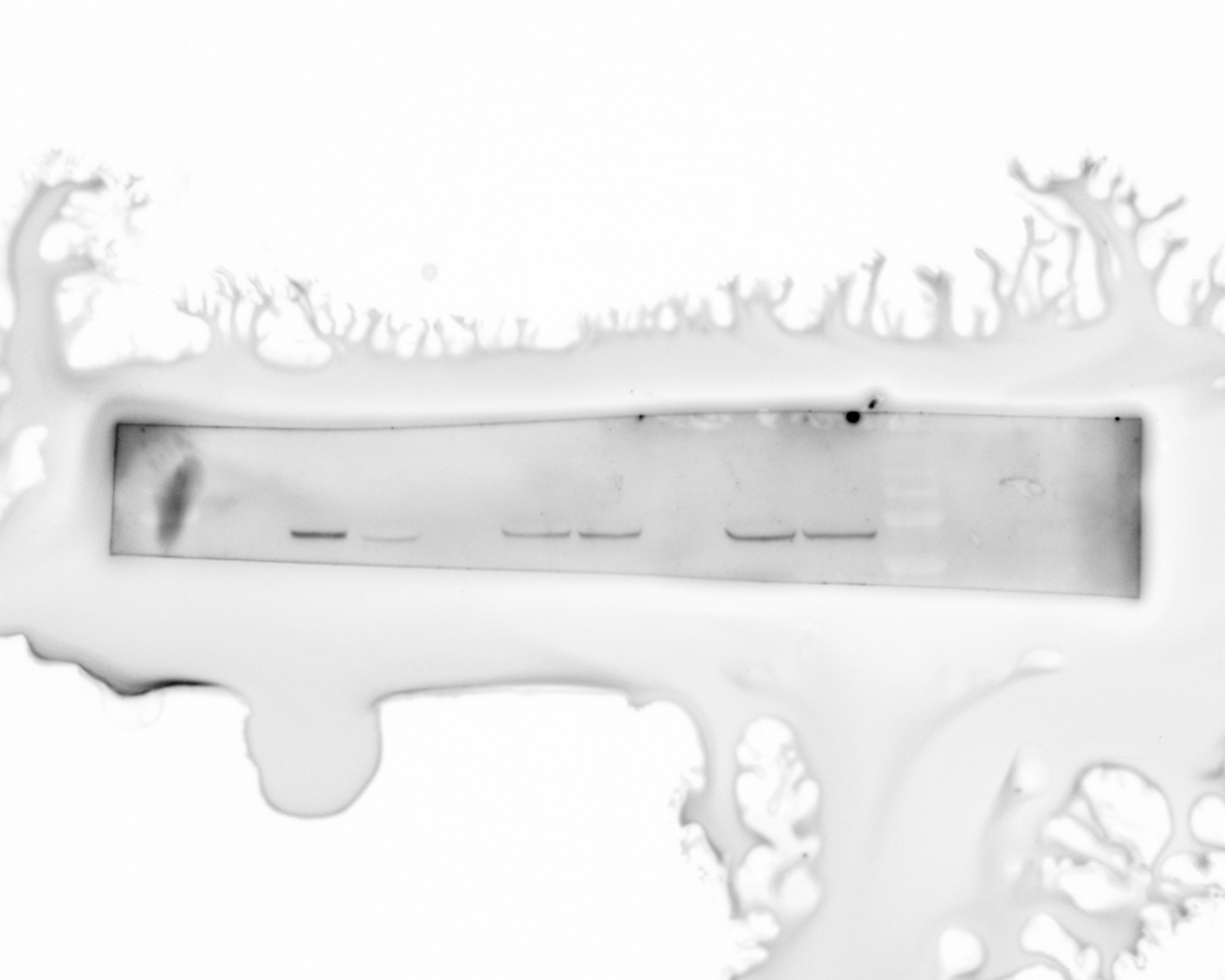

Supplement: Source data 1. [file elife-82568-data1.zip › Source Data files/Raw blot images/Figure 5/Figure 5d anti-SYT1.gif]

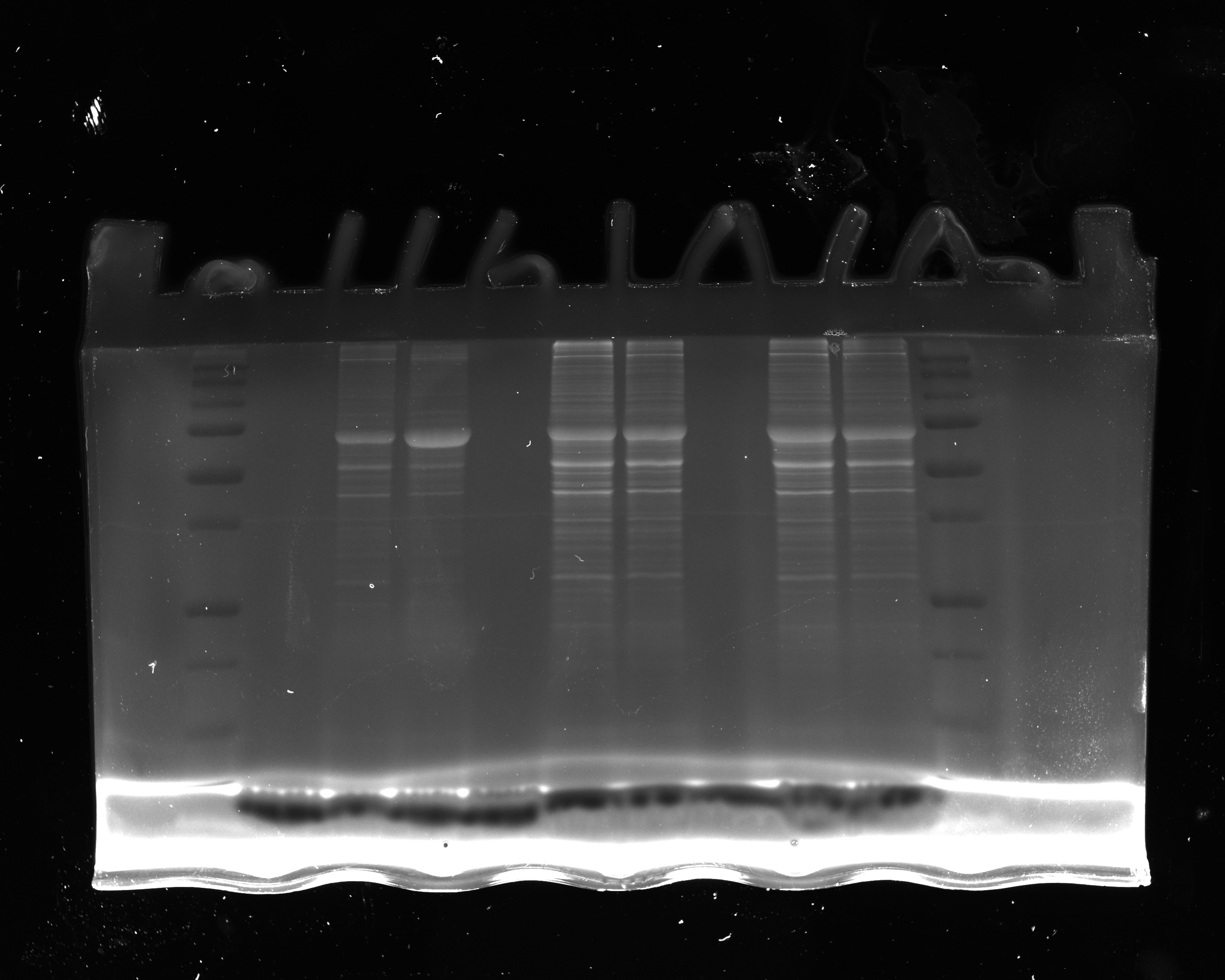

Supplement: Source data 1. [file elife-82568-data1.zip › Source Data files/Raw blot images/Figure 5/Figure 5d TCE load ctrl.gif]

Figure 5D

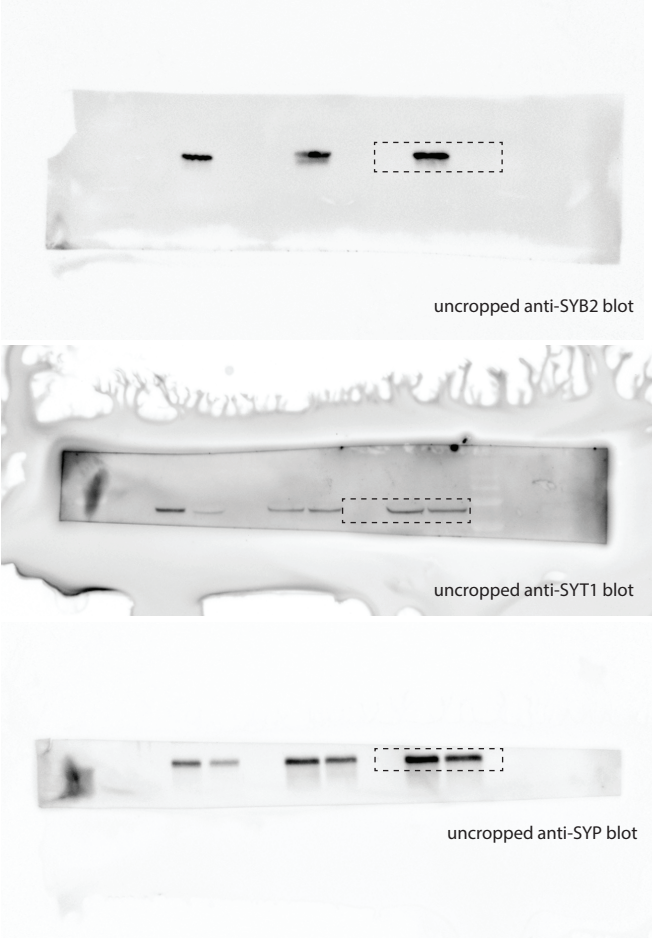

Figure 3 - figure supplement 1A

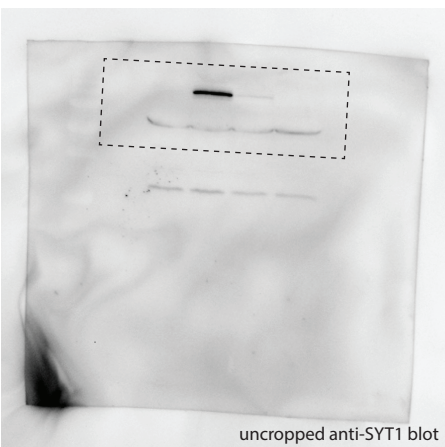

Figure 3 - figure supplement 1B

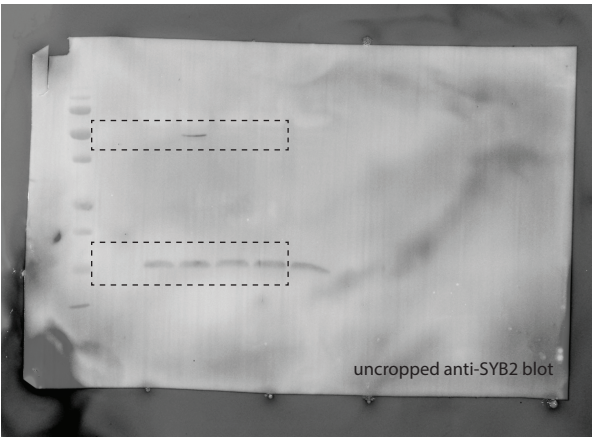

Supplement: Source data 1. [file elife-82568-data1.zip › Source Data files/uncropped blots with labels.pdf]
